# Books and Magazines

**Published:** 1904-03

**Authors:** 


HOW TO ATTRACT AND HOLD AN AUDIENCE. A Popular Treatise on the Nature, Preparation and Delivery of Public Discourses. By J. Berg Esenwein, A. M., Lit. D., Professor of the English Language and Literature in the Pennsylvania Military College. Hinds & Noble, Publishers, New York City. Price, $1.00, cloth, boards. 270 pages.

This little book will be found useful and interesting by all who are ever called on to speak. It maybe a real help to the bashful and inexperienced speaker. We fail to see how it will help any one to "attract an audience," however.

---

THE INTERSTATE MEDICAL JOURNAL for January, 1904, is a valuable book of over 100 pages. It is devoted to a review of medicine in all its branches for the year 1903, and an exposition of its progress. Many of the articles are very able and interesting. Amongst its contributors are Myers, Bartlett, Tanssig, Carl Fisch, Ehrenfest, Friedlander, Engman, Sauer and other well known writers. Dr. O. F. Ball, managing editor, is to be congratulated upon volume XI, No. 1.

---

Nose and Throat for the General Practitioner. By George L. Richards, M. D., Fellow American Laryngological, Rhinological and Otological Society; Fellow American Otological Society; Associate Editor Annals of Otology, Laryngology and Rhinology; Otologist and Laryngologist Fall River Union Hospital, Fall River, Mass. Price, $2.00. Published by International Journal of Surgery Co., New York.

This little work does not enter into a discussion of the theory or the minutia of the subjects treated. It contains just so much of detail as will be of practical use to the general practitioner of wide experience, and is prepared especially for him, describing only such instruments and apparatus as he should possess.

In the matter of illustrations the work is decidedly deficient. We are loath to make this criticism, for the text is, on the whole, unusually clear and concise, and will be of benefit to those in need of such a work.

